# Neither critical shoulder angle nor acromion index were related with specific pathology 20 years later!

**DOI:** 10.1007/s00167-021-06602-y

**Published:** 2021-05-19

**Authors:** Hanna C. Björnsson Hallgren, Lars Adolfsson

**Affiliations:** Department of Orthopaedics in Linköping and Department of Biomedical and Clinical Sciences, Linköping University, S-581 85 Linköping, Sweden

**Keywords:** Shoulder, Critical shoulder angle, Acromion index, Long-term follow-up, Gender

## Abstract

**Purpose:**

The critical shoulder angle (CSA) and the acromion index (AI) are measurements of acromial shape reported as predictors of degenerative rotator cuff tears (RCT) and glenohumeral osteoarthritis (GH OA). Whether they are the cause or effect of shoulder pathologies is uncertain since pre-morbid radiographs most often are lacking. The main aim of this study was to investigate if CSA or AI were related to the development of RCT or GH OA after 20 years. A secondary aim was to investigate if the CSA and AI had changed over time.

**Methods:**

In the hospital archive, 273 preoperative plain shoulder radiographs were found of patients scheduled for elective surgery other than cuff repair and arthroplasty. Forty-five images fulfilled the strict criteria published by Suter and Henninger (2015) and were used to measure CSA and AI with two independent assessors. No patient had any sign of OA in the index radiographs or any information in the medical records indicating RCT. After a median of 20 (16–22) years, 30 of these patients were radiologically re-examined with bilateral true frontal views and ultrasound of the rotator cuff. There were 19 men (20 study shoulders) and 11 females (12 study shoulders).

**Results:**

Mean age at follow-up was 56 (32–78) years. There was no correlation between CSA (*r* = 0.02) (n.s) or AI (*r* = − 0.13) (n.s) in the primary radiographs and OA at follow-up. Nor was any correlation found between index CSA (*r* = 0.12) (n.s) or AI (*r* = − 0.13) (n.s) and RCT at follow-up. Mean difference in CSA was − 1.7 (− 10–3) degrees and mean AI difference was − 0.04 (− 0.13–0.09) between the first and the second radiographs, 20 years later. Bilaterally, mean CSA was 32 and AI 0.61 at follow-up.

**Conclusion:**

In this study, no correlation between the CSA, AI and development of OA or RCT could be found. The mean CSA and AI decreased over a 20-year period but the difference was very small. No difference was found between the study shoulders and the contralaterals. These findings question previously reported etiological associations between scapular anatomy and the development of OA or RCT and thereby the use of these calculations as the basis of treatment.

**Level of evidence:**

III.

## Introduction

**Fig. 1:**
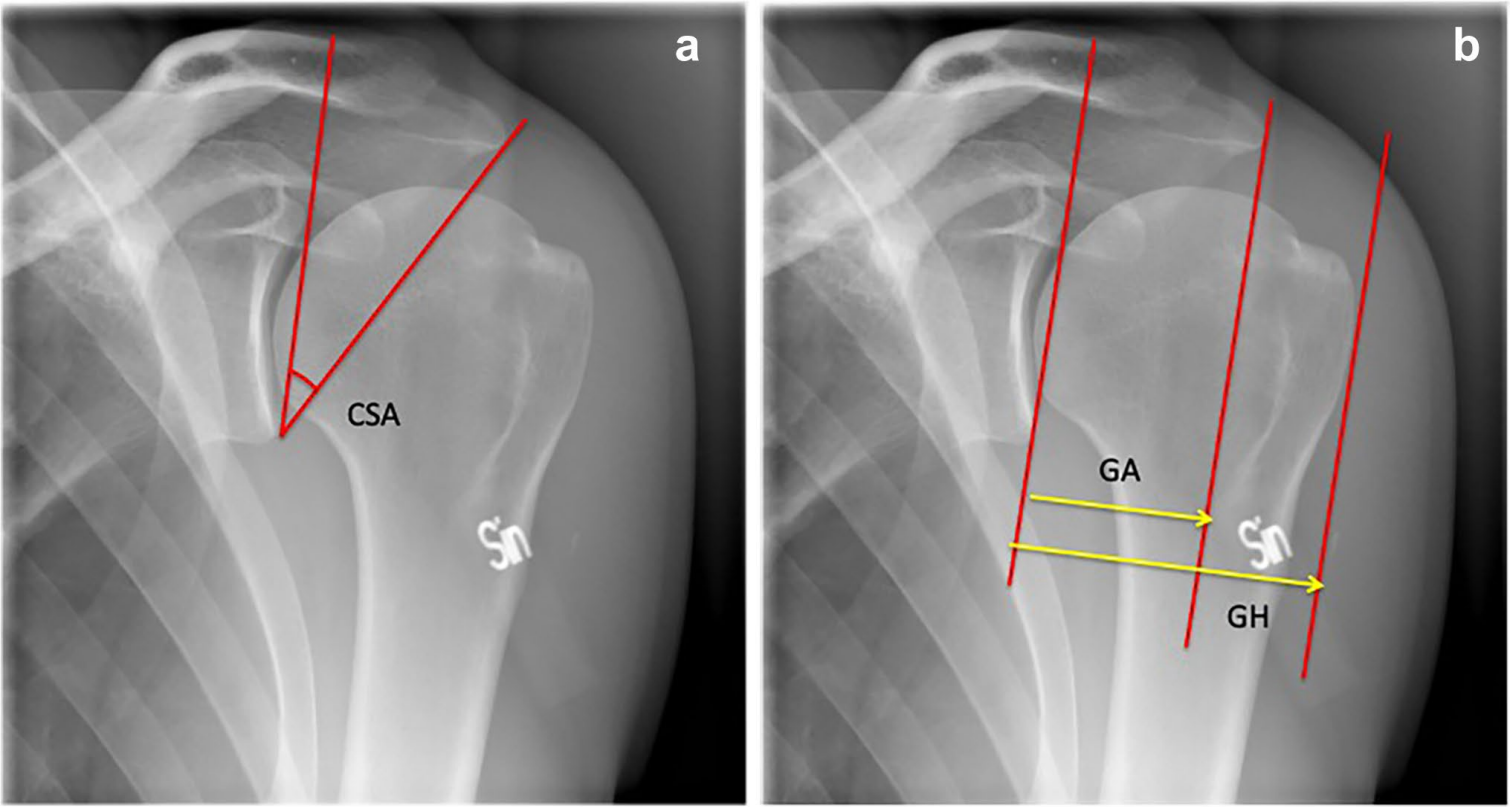
**a** Shoulder radiograph with Suter Henninger A1, schematic drawing of how to measure the critical shoulder angle (CSA). In all the measurements 1 degree of measurement accuracy was used. **b** Shoulder radiograph with Suter Henninger A1, schematic drawing of how to measure the glenohumeral (GH) and glenoacromial distance (GA) used to calculate the acromion index (AI = GH/GA). In all the measurements 2 decimals of accuracy was used

The acromion anatomy has been described to influence, or even elicit, development of different shoulder pathologies. Lately two measures, critical shoulder angle (CSA) and acromial index (AI), first described by Moor et al. [19] and Nyffeler et al. [21] have been found associated with rotator cuff tears (RCT) or glenohumeral osteoarthritis (GH OA) [2, 7, 8, 11, 14, 19, 21, 23, 24, 28]. It has also been suggested that CSA and AI values, in combination with established risk factors such as trauma and age, may predict the integrity of the rotator cuff [20, 26]. The CSA and the AI are coronal plane measurements describing lateral acromial offset relative to the humeral head and glenoid inclination (CSA only) (Fig. 1a, b). In theory, a lateral acromial extension with a CSA above approximately 35° and a high AI (> 0.74), creates a deltoid muscle vector that potentially could cause excessive shear forces in the glenohumeral joint, increased load on the superior rotator cuff tendons and thereby an increased risk of rotator cuff tears [31]. On the contrary, less lateral acromial offset, a CSA below approximately 30° and a smaller AI would theoretically result in larger compression forces in the glenohumeral joint and thereby increase the risk of glenohumeral osteoarthritis (GH OA) [19]. Biomechanical data further support that force vectors acting on the glenohumeral joint are affected by the CSA [8]. The measurements are addressed frequently in scientific articles and possibly also used in clinical practice, but there are still several remaining controversies. It is unknown if differences in acromial and glenoid shapes are a cause or a result of the respective pathologies since pre-morbid radiographs and longitudinal data have been lacking [26, 28]. Published studies have analysed radiographs of numerous patients with already manifest rotator cuff disease or GH OA [12, 28]. Other important considerations are the quality of radiographs and reliability of acromial measurements. True anteroposterior (AP) radiographs are needed for the calculation of CSA and AI as described by Nyffeler et al. [21] and Moor et al. [19]. Interobserver reliability for CSA and AI has been reported to be excellent, but a small deviation from a true AP view affects the CSA since the accuracy of CSA depends on the spatial relationship between the scapula and the radiograph beam, which varies with patient position and posture [11, 12, 16, 17, 26]. To prevent inaccurate CSA, Suter and Henninger (SH) presented a classification of valid AP radiographic views, based on 3-dimensional computed tomography reconstructions of the scapula [26]. No correlation has been found between CSA, gender and side but this is based on cadaveric shoulders without pathology [26].

The hypothesis of this study was that the development of RCT or GH OA would be related to CSA and AI values in premorbid images. According to previous literature, CSA and AI can be expected to stay unchanged over time, be unrelated to gender and similar on the affected and contralateral sides [6, 26]. The main aim of this study was therefore to examine if CSA and AI could predict the development of RCT or GH OA during a 20-year period. Secondary aims were to investigate if the CSA and AI had changed over time and if they differed between shoulders and gender.

## Methods and material

### Study design and selection criteria

This is a study with longitudinal data collected from a cohort of patients operated at our orthopedic department 20 (range 16–22) years ago. The study was performed in accordance with the ethical standards of the Helsinki Declaration and was approved by the regional ethical committee in Östergötland, Sweden DNR 2013/330-31. Informed written consent was obtained from all study participants. Preoperative shoulder radiographs had been taken because of shoulder instability or other disorders (Table 1). Rotator cuff pathology and GH OA had been excluded by radiographs and arthroscopy. Inclusion criteria for the study were an AP shoulder radiograph taken during the years 1995–2002 with projections amenable for determining AI and CSA according to the Suter-Henninger criteria and a shoulder condition not suspected to have affected acromial or glenoid shape. Exclusion criteria were signs of GH OA or rotator cuff arthropathy in the index radiograph, a documented rotator cuff tear in the patients’ medical and surgical records or any information indicating arthritic joint disease. In the hospital archive, 273 non-digital, plain film, shoulder radiographs with AP views of eligible patients were found. The patients had sought care due to instability, SLAP injury, frozen shoulder or subacromial bursitis and preoperative radiographs had been taken before their arthroscopic shoulder surgery. The patients’ pre-and peri-operative medical records were reviewed for diagnosis and information on their rotator cuff status. At the time, an operation-protocol in which the surgeon had described all the pathological findings and the surgical procedure was used in all shoulder arthroscopies.

**Table 1:** Background variables, initial CSA, AI values and diagnosis of the study patients *n* = 30 with *n* = 32 study shoulders

At index examination	Study shoulders *n* = 32 Patients = 30
M/F sex, (*n* = patients)	19/11
SH A1/C1* (*n* = shoulders)	16/16
CSA mean (min–max)	34 (29–39)
AI mean (min–max)	0.65 (0.48–0.76)
Diagnosis	
Subacromial pain/stiffness (*n*)	17
CSA mean (min–max)	35 (29–39)
AI mean (min–max)	0.67 (0.55–0.76)
Instability (*n*)	15
CSA mean (min–max)	32 (29–35)
AI mean (min–max)	0.62 (0.48–0.71)

Measurements at index radiographs and initial diagnosis

*Suter-Henninger classification of a true antero-posterior radiograph

### Radiographic assessment

All images were assessed independently by two assessors (HH, LA), according to the strict SH criteria for a true AP view [26]. Forty-seven images in 45 patients fulfilled the criteria with no double contour of > 50% of glenoid height or an inverted teardrop pattern at the upper glenoid rim (Fig. 1a, b), and no signs of GH OA or RCT [26]. The same assessors then independently measured CSA and AI according to Moor et al. and Nyffeler et al. [19, 21] twice with 2 weeks interval. The original, plain, radiographs were also re-examined and measured a third time, 3 months after the digitalized images had been examined, with the assessors blinded of the first measurement results. The CSA was measured with a protractor using one line connecting the superior and inferior border of the glenoid fossa and another line connecting the inferior tip of the glenoid with the most inferior-lateral point of the acromion in true AP views. Lines for calculating AI were drawn on the same images with one line connecting the superior and inferior extensions of the glenoid fossa, and two additional parallel lines, one at the lateral extension of the acromion and one at the most lateral aspect of the humeral head (Fig. 1a, b).

### Follow-up

**Fig. 2:**
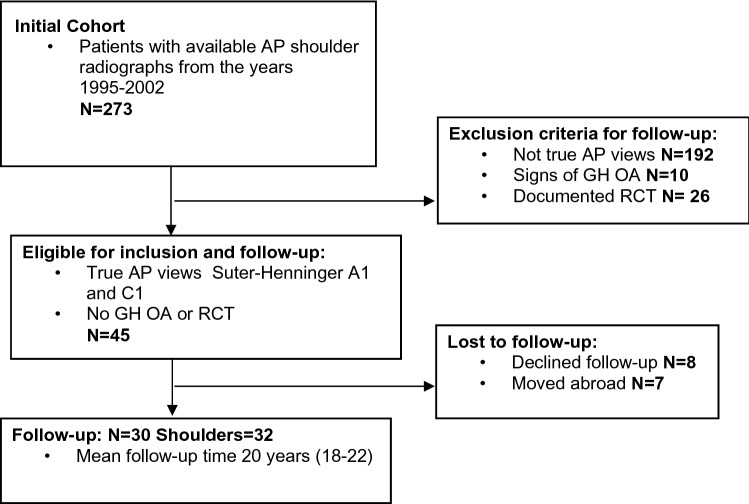
Flowchart of the included patients. *GH OA* glenohumeral osteoarthritis, *RCT* rotator cuff tear

Of the 45 patients, 30 were possible to re-examine after a median of 20 (16–22) years. Seven patients had moved out of the area and eight patients declined to be re-examined due to personal reasons. Two patients had had radiographs taken bilaterally at the initial examination yielding 32 shoulder radiographs available for investigation of longitudinal changes (Fig. 2, flowchart). For comparison between shoulders in the 30 patients, the shoulder unaffected at the index procedure was used as a reference. The radiographs of the 15 patients that declined participation were also examined and did not differ significantly in any measurements. At the follow-up examination, bilateral AP radiographs were taken under fluoroscopic control to obtain true frontal views as recommended by Moor et al. and Suter et al. [19, 26]. Using digital tools, CSA and AI were then calculated from these digital, AP radiographs in the same way as on the initial images with blinded, repeated measurements. Degrees were measured without using decimals. In addition, the images were assessed for GH OA classified according to Samilson and Prieto [22] and cuff tear arthropathy (CTA) according to Hamada [10]. Bilateral ultrasound examination of the rotator cuff was performed by an experienced orthopedic shoulder consultant (HBH) who classified the rotator cuff tendons as intact, or with partial or full-thickness tears. The patients were also asked if they had experienced a significant trauma or new surgery during the follow-up time (Table 2). All follow-up examinations, radiographs, ultrasound and questionnaires included both shoulders.

**Table 2:** Follow-up data of all study patients including, radiology, clinical and ultrasound examinations bilaterally

Follow-up examination	Study shoulders *n* = 32 Patients = 30	Contralateral shoulders *n* = 28 Patients *n* = 28
Age, years at follow-up, mean (min–max)	56 (32–78)	–
M/F sex, (*n*)	19/11	–
Trauma during follow-up period yes/no	2/32°	1/28°
Surgery during follow-up period yes/no	4/32°	2/28°
Pain and/or other symptom yes/no	6/32°	6/28°
Constant-Murley score mean (min–max)^€^	81 (34–100)	84 (25–100)
Radiology*		
SH A1/C1	16/16	10/16
GH OA SP^#^		
0	20	25
1	9	0
2	3	1
CTA Hamada^##^		
0	28	22
2	1	1
3	2	2
4	1	1
Rotator cuff status°		
Intact	21	18
Partial-thickness tear	0	2
Full-thickness tear	10	7
CSA mean (min–max)	32 (26–38)	32 (27–38)
CSA diff mean (min–max)**	− 1.7 (− 10–3)	–
AI mean (min–max)	0.61 (0.49–0.72)	0.61 (0.47–0.71)
AI diff mean (min–max)**	− 0.04 (− 0.13–0.09)	–

°Clinical and ultrasound examination *n* = 31 study shoulders and *n* = 27 contralateral shoulders, one patient denied clinical follow-up

^€^Constant-Murley score 0–100

*Two radiological examinations of the contralateral shoulders could not be used for measurements *n* = 26. One patient was operated with osteotomy of the glenoid and 1did not fulfill the Suter-Henninger (SH) classification of a true antero-posterior radiograph

**Difference in CSA and AI between first and second radiograph examination

^#^Glenohumeral osteoarthritis according to Samilson-Prieto 0–4

^##^Cuff tear arthropathy according to Hamada 1–5

At the final follow-up, the cohort consisted of 19 men (20 shoulders) and 11 females (12 shoulders) with a mean age of 56 years (32–78). Patients were divided into two groups based on their initial diagnoses, anterior instability or subacromial pain/frozen shoulder (Table 1). Fifteen patients had an arthroscopic anterior stabilization at the index surgery, and two of these had undergone a reoperation with another stabilizing procedure during the follow-up period. Three patients had had a frozen shoulder that was assessed arthroscopically and then mobilized during anesthesia. Fourteen patients had had subacromial pain without any signs of a rotator cuff tear and were operated with arthroscopic subacromial decompression (ASD) including joint inspection, resection of the subacromial bursa and a minor anterior/inferior acromioplasty. In no case, a lateral acromial resection was done. None of the patients had any signs of OA at the arthroscopy or in the index radiographs. During the follow-up period 1 of the patients with a stiff shoulder had undergone a second arthroscopic release and another patient an open cuff repair. In the contralateral shoulders two patients had been operated with an anterior ASD during the follow-up period (Table 2).

### Statistical analyses

Descriptive statistics were used to describe baseline and follow-up characteristics and are expressed as median or mean and range of minimum and maximum as applicable. Using the available data, a power analysis was made based on the assumption that a CSA larger than 35 degrees would predict the development of rotator cuff rupture. With a two-tailed 95% level of significance, an equal assumption of likelihood between those with CSA above or below 35° and 80% power, a sample size of 204 patients would have been needed. This was based on the findings in the present cohort in which we found that 60% of the patients who had developed a rotator cuff rupture had a CSA below 35°. Because of the smaller cohort size in the present study, group comparisons were not made and only descriptive data and statistics used. Correlations were investigated with the use of Pearsons product-moment correlation coefficients (*r*). Values of less than 0.25 indicate a weak, correlation; values between 0.25 and 0.49, a mild correlation; values between 0.50 and 0.75, a moderate correlation; and values larger than 0.75, a good correlation [27]. *P* values < 0.05 were considered statistically significant. The IBM SPSS Statistics for Windows, Version 24.0. Armonk, NY: IBM Corp. was used for all statistical calculations.

## Results

In the cohort of 30 patients, the index radiographs were classified as 16 A1 and 16 C1 according to the SH system. The two categories (A1 or C1) were equally distributed between the two diagnostic groups both at the index investigations and at follow-up (Tables 1 and 2). At the index time point, the mean CSA was 34 (29–39) and mean AI 0.65 (0.48–0.76) in the whole cohort. (Table 1). In all the measurements, the intra- and inter-rater agreement between the two assessors was perfect within one degree of measurement accuracy.

At follow-up 12 patients had developed radiological signs of GH OA and ten patients had a verified posterosuperior full-thickness RCT. Four of the ten patients with rotator cuff tears also had radiological findings of cuff tear arthropathy (CTA) (Table 2). No correlation could be found between the CSA (*r* = 0.02) (n.s) or AI (*r* = − 0.13) (n.s) in the primary radiographs and GH OA at follow-up. Nor could any correlation be found between index CSA (*r* = 0.12) (n.s) or AI (*r* = − 0.13) (n.s) and presence of RCT or CTA at follow-up.

At follow-up the mean CSA was 32 (26–38) and mean AI was 0.61 (0.49–0.72). Between the first and the second radiographs, 20 years later, both values had decreased. The mean difference in CSA was −1.7 (− 10–3) degrees and the mean AI difference − 0.04 (− 0.13–0.09). When comparing with the contralateral shoulders at follow-up, a similar reduction of the measured CSA and AI was noted and there was no side-difference, CSA was 32 and AI 0.61 bilaterally (Table 2). Men had a smaller mean CSA both at the index examination and at follow-up 32.6 (29–36) and 31.7 (25.5–38), respectively, than women who had 35.2 (31–39) at the initial and 32.1 (25.5–36.8) at the final examination. The mean difference between the first and second images was − 1 (− 4.7–3) in men and − 3.1 (0 to − 9) in women. Only a minor change in AI between the two examinations was seen, a reduction of 0,04 for both men and women. CSA and AI in the patients that had undergone an anterior acromioplasty at the same time as the index arthroscopy had decreased less (mean − 1.4°, − 0.03) compared with the rest of the cohort (− 1.7°, − 0.04).

## Discussion

The most important finding of the present study was that no correlation was seen between the critical shoulder angle (CSA) or acromion index (AI) and the development of glenohumeral osteoarthritis or rotator cuff tear during a mean 20-year observation period. To the authors’ knowledge, this is the first study on the relevance of CSA and AI, that uses premorbid data and long-term follow-up. The present findings question previously reported etiological associations between scapular anatomy and the development of RCT’s and GH OA [2, 7, 8, 14, 21]. The studied group was, however, relatively small and underpowered to demonstrate a statistically significant difference. An association might still exist, undetected by the present study, but it appears likely that it in that case would be relatively weak.

Most previous studies reporting an association between CSA, and RCT’s or GH OA have been retrospective cross-sectional studies [2, 5, 7, 12, 13, 16, 20, 21, 24, 30] except for one study with longitudinal data over 4 years that supports our findings by concluding that it seems unlikely that rotator cuff tears are related to CSA [6]. The study compared CSA values in patients with degenerative tears and control subjects with frozen shoulder and found difference of about 2°. A small and likely clinically unimportant difference within measurement errors, as stated by the authors [6].

In a recent retrospective cross-sectional study, CSA was suggested as an objective tool to predict RCTs based on comparing CSA values in patients with degenerative RCT to a control group with subacromial bursitis and tendinitis. The authors stated that AI did not have any significant predictive value [18]. No association between AI and the development of RCTs was also reported by Hamid et al. who compared patients with and without cuff tears [11]. In the present study, the patients treated for pain and stiffness had larger CSA (3°) and AI (0.05) than the instability patients on the index images but 60% of the patients who had developed a RCT had an index CSA below 35°. The etiology of RCTs and GH OA seems more likely multifactorial, and the present study does not support that acromial extension and glenoid inclination are of major importance [15, 16, 24].

Both mean CSA and AI were found to slightly decrease during the follow-up period, which differs from the findings by Chalmers et al., who reported no difference in CSA after 4 years [6]. It has been speculated that acromial anatomy is genetically determined by developmental skeletal features instead of acquired degenerative forms [6, 9]. The findings in the present study cannot corroborate any of these assumptions but if changes could have been attributed to degeneration or bone formation occurring over time, this may have affected the observations [17, 32]. The observed change could perhaps also be related to individual differences in activity and load which over the years might create acromial alterations influencing the CSA and AI, something also suggested by Lädermann [17].

A potential concern is that 14 patients had undergone an anterior acromioplasty at the index surgery since, in a retrospective study, Billaud et al. [3] reported decreased CSA values after this procedure. In the present long-term follow-up, the anterior acromioplasty did, however, not seem to have had this effect since the CSA and AI had decreased less in these patients than in the rest of the cohort. Furthermore, the contralateral, non-operated, shoulders had the same CSA and AI values at the 20-years follow-up, a fact that supports accuracy and that an actual decrease occurred over the years.

Females were found to have a larger mean CSA and AI than males at both the index and at the follow-up examinations and their values decreased slightly more during the observation period. Little is published on gender and CSA and AI but Hamid et al. [11] also found that AI was higher in females than males (0.705 versus 0.682, *p* = 0.01). Suter et al. on the other hand could not find any difference in CSA between males and females in cadavers [26]. It should, however, be noted that the differences found were small and may be related to chance as the groups were small.

 With the radiographic criteria used in the present study a measuring accuracy of 89% can be expected according to Suter-Henninger [26]. With these strict criteria only 23% of the 273 initially retrieved images could be used which significantly limited the number of possible patients to follow-up. In the study by Chalmers et al. [6], using the same criteria, 21% of their images could be used to measure CSA and both these studies confirm that regular shoulder AP views most often do not qualify for CSA measuring. Previous studies, investigating associations between scapular anatomy and development of RCT and GH OA, have most often used radiographs taken without considering scapula positioning and the interpretation of these studies could, therefore, be questioned [2, 7, 8, 13, 17, 18, 20]. In the present study, the CSA and AI followed the same trends, between individuals and over time. This may be related to the fact that we used the strict Suter Henninger criteria to select proper images but may also reflect that the patients in the current series did not have any markedly pronounced glenoid inclinations. The predictive value of AI in relation to the development of RCTs and GH OA has been questioned and CSA has gained popularity with proponents maintaining its superiority [11, 18, 29]. The argument for this is the relevance of the glenoid inclination as opposed to the lateral acromial extension in causing increased load on the rotator cuff tendons or the joint surfaces [30].

Published results are diverging, there is a lack of high-quality evidence and the clinical usefulness of AI and CSA as predictors for future pathology, is uncertain. Still, a lateral acromioplasty has been suggested to prevent the development of RCTs but also to protect cuff repairs by lowering the CSA into the normal range [1, 24]. As long as the deltoid insertion is unchanged, the deltoid resultant force vector also remains unchanged, making the rationale for this procedure difficult to understand and evidence for the effectiveness of the procedure is lacking [26]. The procedure is also potentially harmful as the lateral deltoid origin may be affected and it is even suggested that reduction of the CSA might predispose to OA [4].

This study has strengths and limitations. To our knowledge, it is the first study with long-term longitudinal CSA and AI data, intraoperative recordings confirming the status of the rotator cuff at the index timepoint and with bilateral images, ultrasound and clinical data after about 20 years. The best-documented criteria for the selection of index images were used, and all available examinations that fulfilled the criteria were included, limiting selection bias. Two independent assessors did repeat measuring and the follow-up images were taken under fluoroscopic control, standardized to avoid measuring errors. The initial radiographs and the digitalized images were, however, produced by different methods and it cannot be ruled out that the plain images differed somehow and thereby affected the CSA and AI measurements. Any such effect could, however, not be found and the accuracy of the repeated measurements on the initial images was perfect. The study-cohort was small and underpowered and therefore the statistical analyses should be interpreted with caution and used for formatting new hypothesis and designing new studies. The medical and surgical records were retrospectively studied and did not have completely healthy shoulders initially. According to the theory behind the concept it is, however, unlikely that instability, frozen shoulder and subacromial bursitis would have had any influence on the CSA and AI values at the index images. Fifteen eligible patients could not be examined at the follow-up for different reasons but the CSA and AI indexes were measured on their initial radiographs and did not differ significantly from the rest of the cohort.

The clinical implications of our results are that the CSA and AI measures should be questioned as important prognostic factors for the development of RCT or GH OA and that lateral acromioplasty probably does not prevent RCT’s as suggested [14, 25].

## Conclusion

In this study, no correlation between the CSA, AI and development of glenohumeral OA or RCT could be found. The mean CSA and AI decreased over a 20-year period but the difference was very small. A slightly larger CSA was seen in women but no difference between study shoulders and the contralateral shoulders was seen. Altogether these findings question previously reported etiological associations between scapular anatomy and development of glenohumeral OA or rotator cuff tears and thereby the use of these calculations as a basis of treatment.
